# Supplementary material for: Novel neurofilament light (Nefl) E397K mouse models of Charcot-Marie-Tooth type 2E (CMT2E) present early and chronic axonal neuropathy
Source: bioRxiv. 2025 Feb 6:2025.02.02.636117. Preprint. [Version 1] doi: 10.1101/2025.02.02.636117 (PMC11838447; doi:10.1101/2025.02.02.636117)
Supplement: 1 [file NIHPP2025.02.02.636117V1-supplement-1.pdf]

bioRxiv preprint doi: https://doi.org/10.1101/2025.02.02.636117; this version posted February 6, 2025. The copyright holder for this preprint (which was not certified by peer review) is the author/funder, who has granted bioRxiv a license to display the preprint in perpetuity. It is made available under aCC-BY 4.0 International license.

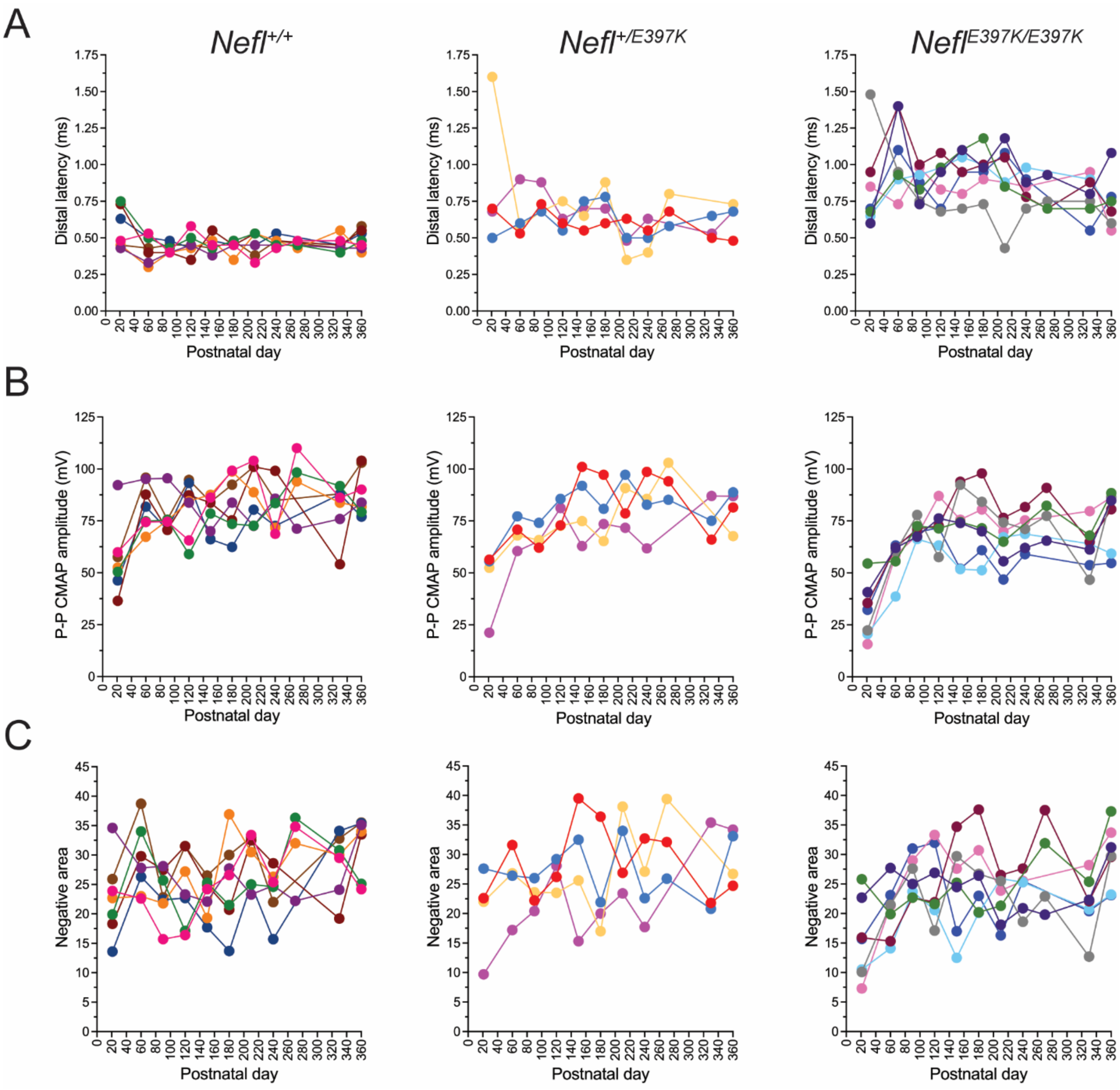

Supplemental Figure 1. Longitudinal study evaluated per mouse. Distal latency, Peak to Peak (P-P) CMAP amplitude and negative area were measured following stimulation of the sciatic nerve and recordings from the gastrocnemius muscle. Wild type (N=7), *Nefl*$^{+/E397K}$ (N=4) and *Nefl*$^{E397K/E397K}$ (N=7) mice were evaluated at P21, P60, P90, P120, P150, P180, P210, P240, P270, P330 and P360 days. Each color represents one mouse (A) Distal latency. (B) P-P CMAP amplitude. (C) Negative area. Ms=milliseconds, mV=millivolts, CMAP=compound muscle action potential, N=number of mice evaluated.

bioRxiv preprint doi: https://doi.org/10.1101/2025.02.02.636117; this version posted February 6, 2025. The copyright holder for this preprint (which was not certified by peer review) is the author/funder, who has granted bioRxiv a license to display the preprint in perpetuity. It is made available under aCC-BY 4.0 International license.

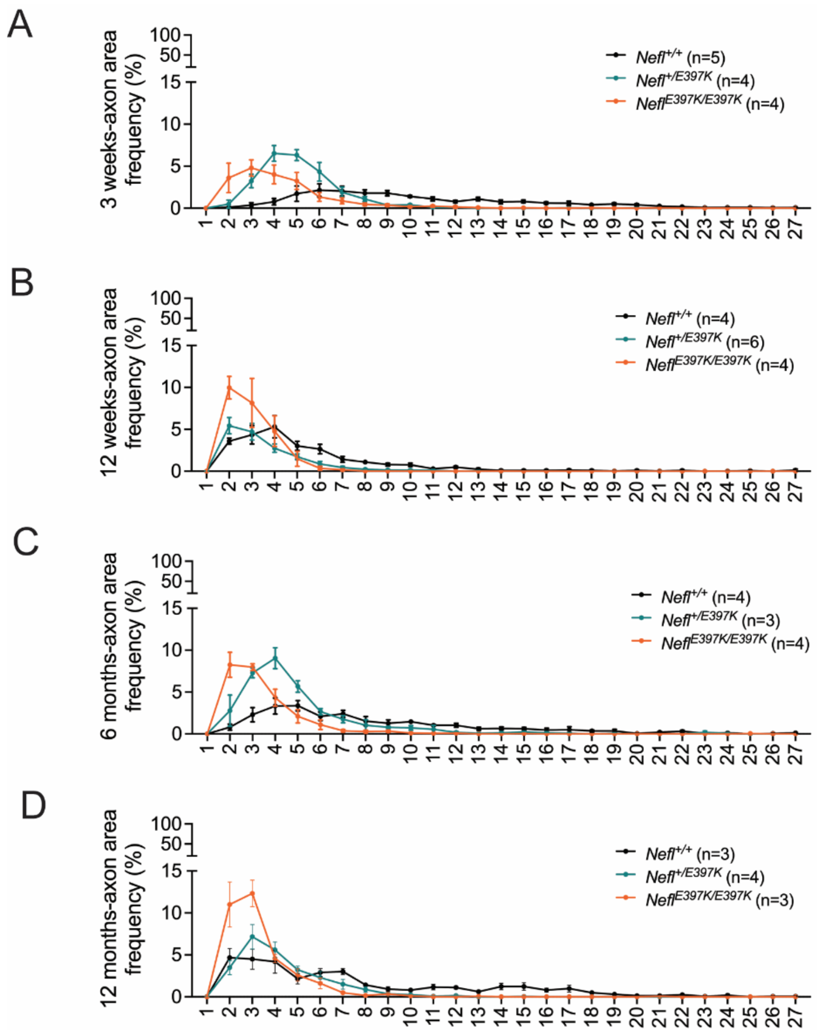

Supplemental Figure 2. Axon area distribution per age group. The sciatic nerve was harvested from wild type, *Nefl*$^{+/E397K}$, and *Nefl*$^{E397K/E397K}$ mice at three weeks, twelve weeks, six months, and twelve months of age. N=number of mice evaluated. Three-week values in μm^2 1=0-1.4, 2=1.4-15.06, 3=15.06-28.71, 4=28.71-42.37, 5=42.37-56.02, 6=56.02- 69.67, 7=69.97-83.33, 8=83.33-96.98, 9=96.98-110.64, 10=110.64-124.29, 11=124.29-137.94, 12=137.94-151.60, 13=151.60-165.25, 14=165.25-178.91, 15=178.91-192.56, 16=192.56-206.21, 17=206.21-219.87, 18=219.87-233.52, 19=233.52-247.17, 20=247.17-260.83, 21=260.83-274.48, 22=274.48-288.14, 23=288.14-301.79, 24=301.79-315.44, 25=315.44-329.10, 26=329.10-342.75, 27=>342.75. Twelve-week values in μm^2 1=0-6.87, 2=6.87-74.59, 3=74.59-142.32, 4=142.32-210.04, 5=210.04-277.77, 6=277.77-345.49, 7=345.59-413.22, 8=413.22-480.94, 9=480.94-548.67, 10=548.67-616.39, 11=616.39-648.12, 12=648.12-751.84, 13=751.84-819.57, 14=819.57-887.29, 15=887.29-955.05, 16=955.05-1022.74, 17=1022.74-1090.47, 18=1090.47-1158.19, 19=1158.19- 1225.92, 20=1225.92-1293.64, 21=1293.64-1361.37, 22=1361.37-1429.09, 23=1429.09-1496.81, 24=1496.81-1564.54, 25=1564.54-1632.26, 26=1632.26-1699.99, 27=>1699.99. Six-month values in μm^2 1=0-3.59, 2=3.59-40.36, 3=40.36-77.13, 4=77.13-113.89, 5=113.89-150.66, 6=150.66-187.43, 7=187.43-224.20, 8=224.20-260.96, 9=260.96-297.73, 10=297.73-334.50, 11=334.50-371.27, 12=371.27-408.03, 13=408.03-444.80, 14=440.80-481.57, 15=481.57-518.34, 16=518.34-555.10, 17=555.10-591.87, 18=591.87-628.64, 19=628.64-665.41, 20=665.41-702.17, 21=702.17-738.94, 22=738.94-775.51, 23=775.51- 812.48, 24=812.48-849.24, 25=849.24-886.01, 26=886.01-922.78, 27=>922.78. Twelve-month values in μm^2 1=0-3.59, 2=3.59-55.54, 3=55.54-107.50, 4=107.50-159.45, 5=159.45-211.40, 6=211.40-263.35, 7=263.35-315.31, 8=315.31-367.26, 9=367.26-419.21, 10=419.21-471.17, 11=471.17-523.12, 12=523.12-575.07, 13=575.07-627.02, 14=627.02-678.98, 15=678.98-730.93, 16=730.93-782.88, 17=782.88-834.84, 18=834.84-886.79, 19=886.79-938.74, 20=938.74-990.69, 21=990.69-1042.64, 22=1042.64-1094.50, 23=1094.50- 1146.55, 24=1146.55-1198.51, 25=1198.51-1250.46, 26=1250.46-1302.41, 27=>1302.41.
